# What influences feeding decisions for HIV-exposed infants in rural Kenya?

**DOI:** 10.1186/s13006-017-0125-x

**Published:** 2017-07-12

**Authors:** Helen M. Nabwera, Joyline Jepkosgei, Kelly W. Muraya, Amin S. Hassan, Catherine S. Molyneux, Rehema Ali, Andrew M. Prentice, James A. Berkley, Martha K. Mwangome

**Affiliations:** 1 KEMRI Centre for Geographic Medicine Research Coast, P.O. Box 230-80108, Kilifi, Kenya; 2 The Childhood Acute Illness & Nutrition (CHAIN) Network, PO Box 43640-00100, Nairobi, Kenya; 3 Centre for Tropical Medicine and Global Health, Nuffield Department of Medicine, University of Oxford, Old Road Campus, Headington, Oxford, OX3 7LF UK; 4 MRC Unit, The Gambia, PO Box 273, Banjul, The Gambia; 5 MRC International Nutrition Group, London School of Hygiene and Tropical Medicine, Keppel Street, London, WC1E 7HT UK

**Keywords:** HIV, Infant feeding, Malnutrition, Decision making

## Abstract

**Background:**

Infant feeding in the context of human immunodeficiency virus (HIV) poses unique challenges to mothers and healthcare workers in balancing the perceived risks of HIV transmission and nutritional requirements. We aimed to describe the decision-making processes around infant feeding at a rural HIV clinic in Kenya.

**Methods:**

We used a qualitative study design. Between March and August 2011, we conducted in-depth interviews (*n* = 9) and focus group discussions (*n* = 10) with purposively selected hospital and community respondents at Kilifi County Hospital, Kenya. These respondents had all experienced of infant feeding in the context of HIV. These interviews were informed by prior structured observations of health care worker interactions with carers during infant feeding counselling sessions.

**Results:**

Overall, women living with HIV found it difficult to adhere to the HIV infant feeding guidance. There were three dominant factors that influenced decision making processes: 1) Exclusive breastfeeding was not the cultural norm, therefore practising it raised questions within the family and community about a mother’s parenting capabilities and HIV status. 2) Women living with HIV lacked autonomy in decision-making on infant feeding due to socio-cultural factors. 3) Non-disclosure of HIV status to close members due to the stigma.

**Conclusion:**

Infant feeding decision-making by women living with HIV in rural Kenya is constrained by a lack of autonomy, stigma and poverty. There is an urgent need to address these challenges through scaling up psycho-social and gender empowerment strategies for women, and introducing initiatives that promote the integration of HIV infant feeding strategies into other child health services.

**Electronic supplementary material:**

The online version of this article (doi:10.1186/s13006-017-0125-x) contains supplementary material, which is available to authorized users.

## Background

Breastfeeding is the backbone of childhood survival initiatives worldwide. However, infant feeding in the context of human immunodeficiency virus (HIV) poses major dilemmas for both carers and health care workers (HCWs), particularly in resource poor settings where safe and affordable infant feeding options are limited [1–3]. Whilst breastfeeding carries some risk of vertical transmission [4, 5], breast milk alone is adequate in meeting the nutritional needs of the majority of infants under 6 months of age [6]. It also provides over half of the energy and nutrient intake of infants over 6 months of age, which can be crucial given the poor nutritional value of many complementary infant foods given in sub-Saharan Africa [7–9]. Breastfeeding is also associated with reduced childhood infections and mortality, as well as better growth and neurodevelopment outcomes [10]. In addition, maternal health is improved through child spacing [10].

For infants born to women living with HIV in resource limited settings, exclusive breastfeeding where infants receive only breast milk in the first 6 months, is recommended by the World Health Organization (WHO), as it reduces the risk of breast milk transmission of HIV 2–4 fold when compared to mixed feeding in the absence of other interventions [11–13]. Mixed feeding where infants under 6 months receive other fluids and/or semi-solids in addition to breast milk, is also associated with reduced HIV-free survival and is therefore not recommended [13]. In relatively affluent urban African settings, trials of replacement feeding with formula demonstrated benefit in terms of HIV-free survival of infants born to HIV infected mothers [14, 15]. However, data from more rural African populations suggest that the use of replacement feeds and/or rapid weaning off breast milk at 6 months of age are strategies that are nutritionally hazardous and detrimental to child survival, with limited benefit of HIV-free survival [16–23]. The reasons for this include poverty, lack of access to clean water and adequate sanitation, and low literacy rates in many rural African settings [7, 24]. With maternal combination antiretroviral treatment (ART) and infant prophylaxis with nevirapine or zidovudine, the risk of vertical transmission in exclusively breastfed infants can be reduced to less than 1% [25–29], and HIV-free survival at 24 months increased to more than 87% [20].

Considerable progress has been made in implementing these interventions for the prevention of vertical transmission in sub-Saharan Africa, with the universal adoption of option B+ by WHO [30]. However, for many countries, reducing the vertical transmission of HIV has remained challenging due to both resource limitations and socio-cultural barriers to exclusive breastfeeding. Exclusive breastfeeding is often viewed as being nutritionally inadequate and harmful for the baby, and exclusive replacement feeding is culturally unacceptable [31–34]. The uptake of combined ART during pregnancy by women living with HIV has also been suboptimal [35].

A cross-sectional health survey conducted in Kilifi County in 2011 showed that although breastfeeding was common, exclusive breastfeeding was not the norm with only 22.4% of infants under 6 months exclusively breastfed [36]. More than 70% of mothers introduced complementary foods by the fourth month of life [37]. These foods included supplemental liquids such as water, fresh cow’s milk and semi-solid feeds such as thin *uji* (maize meal porridge) [38]. These patterns have important implications for vertical transmission. HIV is a major attributable cause of in hospital deaths for children between 6 to 60 months of age admitted to Kilifi County Hospital (KCH) with severe acute malnutrition (SAM) [39]. When we reviewed two and one half years’ worth of routinely collected data from our HIV clinic of 486 HIV exposed and infected infants between 0 and 24 months of age, we found that on at least one visit 19% were severely wasted, 21% were moderately wasted and 18% severely stunted. Only 50% of these mothers reported practising exclusive breastfeeding under 6 months.

The aim of this study was therefore to describe the decision-making processes around infant feeding at a rural HIV clinic in Kenya in order to identify the constraints to the recommended infant feeding practices for infants of women living with HIV.

## Methods

### Study design

The study utilised qualitative methodology using a descriptive-exploratory approach to explore decision-making processes, constraints, knowledge, attitude and practice of infant feeding among carers and HCWs.

### Setting

The study was conducted in Kilifi County Hospital (KCH), a government facility located on the Kenyan coast that serves a rural population of about 270,000. Over 80% of the population in Kilifi County live below the poverty line and rely on rain-fed subsistence farming. It is an area that suffers frequent food insecurity requiring emergency relief operations [40].

In 2014, the overall adult prevalence of HIV in the county was 4.4%, with higher prevalence among women (6.3%) than men (2.3%) [41]. In 2011, the antenatal clinic prevalence of HIV in KCH was approximately 6%, and about 24% of children admitted to the hospital with severe acute malnutrition were HIV infected.

Since 2003, KCH has been providing HIV services including the prevention of vertical transmission interventions and early infant diagnosis (EID). All nurses in the antenatal, postnatal and family planning clinics are trained in prevention of vertical transmission and almost all mothers attending KCH clinics are tested for HIV (> 90%). Women living with HIV are advised to give birth at a health facility and to register their infants for care soon after delivery at the KCH HIV clinic or the nearest health facility providing prevention of vertical transmission and EID services. In the KCH HIV clinic, 65% of the infants do not complete the 18 month follow-up period, with 43% of drop outs occurring within 2 months of enrolment [42].

At the time of this study, the national HIV infant feeding guidance was changing in line with the 2010 WHO guidance [13]. The prior recommendation had been exclusive breastfeeding in the first 6 months or replacement feeding only if it was acceptable, feasible, affordable, sustainable and safe (AFASS), and continuation of breastfeeding until the age of 12 months with the introduction of complementary feeds, if conditions of replacement of breast milk were still not met [43]. In addition, women living with HIV who were already on combined antiretroviral treatment (ART) were to continue with this, but for those who were not, it was recommended that they start zidovudine at 28 weeks’ gestation then, combine ART (zidovudine, lamivudine and nevirapine at the onset of labour with the continuation of zidovudine and lamivudine only up to 7 days [43]. For the infant nevirapine was to be administered within 72 h of birth plus lamivudine and zidovudine for 6 weeks [43]. This was subsequently revised in line with the WHO recommendations for the continuation of combined ART in the mother and nevirapine in the infant for the duration of breastfeeding [13].

### Study sample

In order to gather a range of views on experiences of infant feeding in the context of HIV, male and female respondents from both the hospital and community were purposively sampled. This included both carers and health care providers. Hospital respondents came from a range of departments within the hospital where nutritional counseling was offered to women living with HIV. These departments included the HIV clinic, maternal and child health clinic (MCH) antenatal (ANC) and postnatal clinics (PNC), and the paediatric wards. Respondents from the community in the hospital catchment area were selected to supplement the information from carers and health care workers; and to further explore community perceptions of infant feeding practices in the context of HIV. These comprised Kenya Medical Research Institute (KEMRI) Community Representatives (KCRs) who are carefully selected members who are typical of those communities and represent the geographical areas in which a diverse range of health studies are conducted [44].

### Data collection and analysis

**Fig. 1:**
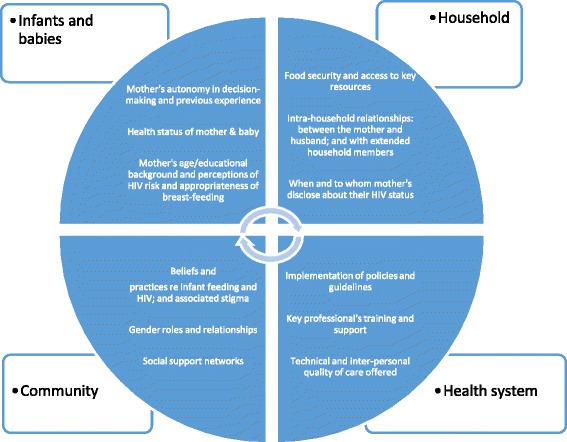
Conceptual framework of factors that influence infant feeding practices among women living with HIV rural Kenya

A conceptual framework was developed based on the literature available on the factors that may influence infant feeding in low income countries (Fig. 1) [1, 2, 45]. We undertook structured observations of infant feeding counselling sessions at the KCH outpatient clinical areas including the HIV clinic. The information from these observations were then used to refine the conceptual framework which in turn helped guide the ideas to be explored in the focus group discussion (FGD) and in-depth individual interview (IDI. The information was also used in the analysis to contextualise some of the experiences that the respondents shared about infant feeding counselling sessions. These guides covered a range of topics including factors influencing infant feeding decision making and constraining adherence to infant feeding recommendations in the context of HIV. The observation tools and guides for the carers were translated from English to Kiswahili by an experienced translator, according to the principles of the WHO translation protocol [46]. An additional file shows the Kiswahili version of the observation tool (see Additional files 1 and 2). All interviews and group discussions were conducted by experienced facilitators and were undertaken in English, Kiswahili or in the local dialect, Kigiriama. Sessions were audio-recorded and additional notes of the interview dynamics and environment taken. In total 9 IDIs and 10 FGDs were undertaken. Interviews were transcribed verbatim and, when required, translated into English. Data were managed using NVivo (QSR International Pty Ltd. 2012) [47].

Data were analyzed using a thematic framework approach [48], which incorporated elements from the conceptual framework. This entailed extensive familiarization of the transcripts by HMN and JJ who independently immersed themselves in the data, made summary sheets of each transcript and took related notes of key emerging themes. These preliminary themes were then discussed amongst the lead authors HMN, JJ, KWM and MKM, who had independently reviewed two transcripts. Through a process of careful discussion, comparisons of emerging themes and emerging consensus, a coding scheme was developed. This coding scheme was used to code the data in NVivo. The coded data were drawn upon to develop charts of key data to support categorization and exploration of emerging issues and comparison across interviewees [48].

## Results

### Family structure and dynamics

Mothers often lived with their extended families. In this context women living with HIV who had not disclosed their status to their family members found it difficult to undertake feeding practices that departed from the norm (such as sustaining exclusive breastfeeding for 6 months), even when they were motivated to do so. The practice of “communal” caring of young children in such homes increased the chances of another family member introducing other feeds to the baby in the mother’s absence. For instance, a young mother’s insistence on exclusive breastfeeding where this was contrary to the advice or instructions given by the senior woman risked damaging family relationships. The younger woman risked being perceived as stubborn, disrespectful, or too lazy to prepare “real” food for the baby.

*. . . for example you have gone to fetch water from a place far away and you live with your mother in-law and leave the child with her, when you come back the child . . . has been given porridge . . . (Female HCP, FGD, HIV Clinic, P6).*

Mothers often lacked autonomy in the decision-making processes around infant feeding in the context of HIV, as children in the study community were generally perceived as “belonging” to the father. Therefore, although fathers rarely attended infant feeding counseling sessions and had limited knowledge of the recommendations, they had to be consulted and their consent obtained for child health related matters, including decision making on new approaches to child care or feeding. This was particularly pertinent whenever financial resources were required, for example for mothers who had opted for exclusive replacement feeding.

*In our set up a mother is supposed to breastfeed and if she opts for replacement feeding this can be an issue and [there is] the “mwenye culture”* [child belonging to the father] *if you are married you are not supposed to decide without your husband . . . (Female HCW, FGD, P2)*

Conversely, when mothers opted to practice exclusive breastfeeding they did not feel empowered to go against their husbands’ instructions if he insisted on additional feeds. This contributed to mixed feeding through the introduction of complimentary foods such as porridge, even when the mother was aware of the increased risk of HIV transmission associated with this.

*But some [fathers] even tell us to give porridge to boost the baby’s weight or to stop the baby from crying to allow both of you to rest . . . (Female HCP, FGD, ANC, P6).*

In some instances, mothers suffered adverse consequences as a result of adhering to recommended infant feeding practices against the will of their spouses, such as being abandoned by the husband or receiving verbal abuse, leading to strained marital relationships.

To overcome challenges related to adhering to recommended feeding practices, mothers often disclosed their HIV status to particular close relations such as their birth mothers or partners, in order to get some support for their infant feeding choices.

*. . . She knows [her older daughter knows about her HIV status]. . . She was sent to get water for my baby [by her sister] and she refused, I was told by a doctor not to give water to the child. . . its good if you would explain to somebody your condition and she assists you. . . (Female HCP, FGD, HIV clinic, P6).*

However, not all mothers were able to disclose their status, particularly to their husbands. Some mothers therefore developed strategies to support their infant feeding choices for example, by framing those choices as general health advice that is given to all mothers at the health facility.

*So we say it is advice that we (all mothers) were given at the clinic. . . (How infected mothers defend their choice to exclusively breastfeed their infants) (Female HCP, FGD, HIV clinic, P3).*

### Uncertainty of child’s HIV status

Women living with HIV reported that the decision to breastfeed or give replacement feeding was primarily based on their desire to protect their child from HIV infection. At the time of the study, the turnaround time for confirmatory test results for infants’ HIV status varied from one to six months. Mothers reported that they considered breastfeeding because they were not sure of their infants’ HIV status soon after birth and that they would have considered not breastfeeding if they had been sure that their child was not infected from birth.

*I plan to breastfeed my baby since it will be quite difficult to know the child’s HIV status right at birth . . . .I would not breastfeed him had I had the surety that, he is not HIV infected. It would be unfair if I don’t breastfeed him only to later realize that he is [after all] HIV infected . . . (Female HCP, IDI, HIV clinic, P2).*

*As per the counseling, I was informed that, it wasn't possible to know whether a child is infected on his very first day. I thus opt to breastfeed the baby since it isn’t possible to know the child's fate. I will exclusively breastfeed the baby for the first six months while awaiting the results. I will stop the breastfeeding when the results turn out negative. (Female HCP, IDI, HIV clinic, P2).*

In contrast, but with the same intent, other mothers coped with this uncertainty by opting for exclusive replacement feeding over exclusive breastfeeding despite it being unaffordable and unsafe in this rural setting. This was because they worried that other family members who were unaware of the dangers of mixed feeding in the HIV context might give other feeds to the child increasing the risk of vertical transmission.

*It’s because I am living with HIV and was given the option of either to exclusively breastfeed for six months or not to breastfeed at all. But I personally decided not to risk by breastfeeding so I didn’t . . . (Female HCP, FGD, MCH, P1).*

*Mothers living in the villages choose not to breastfeed at all because they fear someone giving the child things like water . . . (Female HCP, FGD, ANC, P1).*

### Interaction with the health system

Contact with the health facilities especially during antenatal care visits was reported by carers to have been highly influential in their infant feeding decision making.

*I started clinic when I was three months pregnant. I had no idea of the PMTCT program but upon enrolling for care, I met X and her colleagues who counseled me and I decided that I shall exclusively breastfeed my child for the first six months . . . (Female HCP, IDI, HIV Clinic, P1).*

This was particularly true for multiparous women living with HIV who were keen to continue with infant feeding practices that had worked for them with previous infants, whether that was exclusive breastfeeding under 6 months or exclusive replacement feeding.

*I will continue (exclusively) breastfeeding this one . . . I will follow the advice given as with the other child who eventually turned out to be negative and for this one I will also do that . . . (Female HCP, FGD, HIV Clinic, P6).*

Although carers reported the importance of health system contact on their choices, several health workers felt that this did not impact on mothers’ infant feeding decisions as many mothers within the clinic still practiced mixed feeding. This observation suggests that socio-economic and cultural considerations can overwhelm stated intentions.

Support from peers also provided mothers with more information about the infant feeding recommendations, apparently influencing infant feeding choices. Women living with HIV enrolled in a mother-to-mother (M2 M) support group reported being better able to adhere to feeding recommendations.

*Before the M2M program, we never had enough time to discuss about the infant feeding. It was just a short time of discussion with either a clinician or a counselor . . . before the M2M, most mothers were mix feeding their babies . . . (Female HCP, IDI, HIV Clinic, P2).*

### Carers’ financial status

The majority of the women living with HIV chose to practice exclusive breastfeeding for infants under 6 months, stated that they did so because it was a cheaper feeding option.

*If you breastfeed the child it helps in reducing the [household] expenses of buying powdered milk until the child is six months, then you can change to cow’s milk . . . (Female FGD, HIV Clinic, P14).*

Financial constraints that were perceived to impact maternal nutritional status were also perceived to impede effective breastfeeding through reducing the quality or quantity of milk.

*Others would like to breastfeed their babies but you find that because of dietary factors they are not able to feed well so they say because I’m not sure if I’m going to be able to feed well for the six months I will rather choose the supplements maybe because they are cheaper at their homes . . . (Female HCW, IDI, P1).*

Job insecurity was linked to financial challenges, reportedly impacting infant feeding decision making because cost of the replacement feeds relies heavily on presence of a stable income over the 6 months period. Where this was not available, or when carers’ situations abruptly changed, this contributed to mixed feeding with consequences for vertical transmission.

*There was a client who opted for replacement feeding and after some time she relocated and I think she got sacked from work or something and she could not afford buying the milk and she went back to breastfeeding . . . (Female FGD, HCW, P3)*

### Policy changes/transition and health care worker constraints

Health care workers (HCWs) commonly reported that the ‘constantly changing policies and guidelines’ impacted on adherence to recommended feeding practices. Some HCWs reported being confused about the guidelines, specifically whether HIV infected mothers should breastfeed their infants or not. This implies the potential for mothers to receive contradictory health messages which could in turn impact on their infant feeding practices. The changing policy landscape was reported to reach HCWs in the more rural or peripheral facilities more slowly than those working in larger or more urban facilities.

*This issue of infant feeding and HIV has brought mixing of issues you find we are well updated here at the [county] hospital but our colleagues, in more remote areas may not be as updated . . . (Female HCW, FGD, P9).*

Health workers identified two challenges with regards to changes in recommended infant feeding practices with implications for maternal feeding choices: i) inadequate training and updates on the recommended infant feeding practices, both in general, and in the context of HIV and; ii) overwhelming workloads resulting in inadequate time for counseling and long waiting times for mothers. These challenges impact on the quality of nutrition advice, counseling and support that HCWs are able to give to the mothers.

*. . . The challenge also comes with the workload so you don’t take time with a patient. You don’t have even time to demonstrate how to prepare the feeds . . . (Female HCW, FGD, P12).*

## Discussion

Our study showed that women living with HIV in this community were constrained from adhering to the infant feeding guidance by three main factors: fear of going against cultural norms of infant feeding as exclusive breastfeeding is alien to this community, a lack of autonomy in the infant feeding decision-making and non-disclosure of HIV status to close family members.

In an era when the infant feeding recommendations on prevention of vertical transmission of HIV for sub-Saharan African mothers is backed by evidence of improved HIV free survival [12, 13, 16, 20, 24], our study showed that the uptake was poor in this rural African community, as in other similar settings [49]. In this paper, we have shown that the uptake of infant feeding recommendations has been hampered by a number of factors. Importantly, we found that the practice of exclusive breastfeeding was uncommon in Kilifi as in many communities in sub-Saharan Africa [50–53]. Therefore mothers who adhered to exclusive breastfeeding were likely to be singled out as being HIV infected [54], inadvertently disclosing their HIV status.

Mothers often lacked autonomy in infant feeding decision-making, despite being counselled on the best options for them. The communal nature of childcare in this rural community posed the risk that another family member would feed the baby on other fluids or solids in her absence. This elevated the risk of vertical transmission and undernutrition in HIV-exposed infants. We found that the family structure and dynamics greatly influenced infant feeding choices and that the decisions were made by dominant family members including mothers in law or husbands, who are rarely targeted in infant feeding or prevention of vertical transmission counseling sessions at health facilities. The role of grandmothers in infant feeding has been widely documented elsewhere in Africa, and their ideas often contradict recommended feeding practices [55–60]. In a study conducted in South Africa in 2004 that predominantly recruited single young HIV infected mothers 25 (63%), for example, 20 (80%) of these mothers who had chosen to exclusively breastfeed had introduced other liquids in the first month of life due to pressure from family members [61]. The majority of the women in the study are financially dependent on their mothers or mothers-in-law and therefore found it a challenge to protect their autonomy on the infant feeding decision-making [61]. This highlights the importance of ensuring that interventions take into consideration the power dynamics within families and communities and therefore incorporate strategies that target those influential ‘others’. Such strategies can include home-based, and more family inclusive counseling sessions. There is also a need to empower mothers with skills to negotiate these power dynamics within the family [56].

Our data also showed that the delays in turnaround time for the results of the infant HIV polymerase chain reaction (PCR) limited the autonomy of some mothers to opt out of breastfeeding, in their attempt to safeguard their HIV PCR negative infants against the continuing exposure to HIV via breast milk. Without these results, they often opted to continue exclusive breastfeeding but then later carried the guilt of transmitting HIV to their infants where positive results were returned. This is consistent with the findings in South Africa in 2004, where infant feeding decision making was reportedly influenced by the desire to protect their infants from risks of vertical transmission [61]. In our context, the use of alternatives to breastfeeding would not be a viable option. Our findings therefore highlight the need for health care workers to utilize a more empathic approach to the issue of non-adherence in their prevention of vertical transmission counseling sessions, recognising the positive intentions and psychological stress that HIV infected mothers undergo in making infant feeding choices. Ultimately this approach may also help to promote adherence to recommended practices [62].

Disclosure of their HIV status sometimes resulted in mothers receiving increased support from friends and significant family members, with positive implications for their adherence to safe infant feeding practices. This has also been reported elsewhere in Africa [63]. Unfortunately, this was not the norm in our setting, as disclosure often had adverse effects on marital relationships resulting in the abandonment and rejection of mothers and their children leaving them destitute. This has been commonly reported in many African settings [63–65]. Women therefore understandably chose not to disclose their HIV status. This led to significant challenges with adherence and families often opted to follow infant feeding ‘norms’ of mixed feeding from as early as 1 month of age.

Undernutrition and HIV often coexist in the context of broader structural factors such as endemic poverty and high food insecurity [66]. In our study, some mothers associated breast milk volume with adequate maternal diet, and many mothers perceived they had milk insufficiency as food insecurity is common in this area [67, 68]. This potentially contributed to many mothers practicing mixed feeding [60]. Sustainable strategies that address food insecurity and poverty among these vulnerable mothers could enhance adherence to recommended infant feeding practices such as exclusive breastfeeding for the first 6 months of life [37].

Beyond decision-making processes at the household/community level, health system related factors were also seen to impact on infant feeding choices by both carers and HCWs. HIV infected mothers expressed the value that they derived from a facility initiated peer group that helped to mitigate the challenges that they experienced with adhering to the infant feeding guidance. Peer support groups can be a very useful strategy for channeling prevention of vertical transmission recommendations to communities whilst providing psychosocial support for HIV infected mothers [69, 70]. Mothers also reported that their interaction with healthcare workers was beneficial in helping them make informed choices and in enhanced adherence to the infant feeding recommendations. This is consistent with findings of a study conducted in South Africa in 1999 [71], where healthcare workers were reported as an important influence on carers’ infant feeding choices. However, our data also showed that mothers of only less than 50% of infants at enrolment to the HIV clinic reported exclusive breastfeeding. This suggests that most mothers did not adhere to the infant feeding counseling in the antenatal and postnatal clinics due to the constraints that we have already stated and in reality, the proportion exclusively breastfeeding may be lower. Although, we were not able to elicit HCW factors associated with non-adherence from the mothers, studies from South Africa found that mothers did not trust healthcare workers and cited inconsistent and confusing advice provided by them [72, 73].

There had been changes to the national PMTCT and infant feeding policies at the time of the study giving greater emphasis to exclusive breastfeeding in the first 6 months, in combination with prolonged use of antiretroviral drugs for mothers and infants [13, 74–76]. The HIV infected mothers we spoke to did not report policy changes as being a barrier to the uptake of infant feeding recommendations because of more immediate pressing constraints. This suggests that irrespective of infant feeding policy changes, underlying factors affecting infant feeding decision-making processes need to be addressed to support mothers’ adoption of new practices, particularly where these deviate from community norms.

Although mothers did not raise direct concerns about infant feeding policy changes, HCWs expressed their discontent at the lack of consistent and timely updates on changes, attributing this to the lack of clarity nationally in technical aspects of MTCT, as well as other facility level challenges such as heavy workload and inadequate resources [77]. This suggests that the implementation of new policies on infant feeding strategies in the context of PMTCT sometimes fails to adequately reach health care workers in rural and remote areas in Africa, which has an impact on the quality of infant feeding counseling [78–80]. There is therefore an urgent need to evaluate innovative cost effective strategies before rolling out new infant feeding and PMTCT national policies. This could be done within a framework of continuing professional development where mentors and trainers interact with the frontline health care workers regularly either remotely or by visiting their work stations to provide ongoing mentorship. This would allow the health workers time to assimilate the new policies whilst reflecting on their infant feeding counseling practices and on how to improve on these skills [81].

A key strength of this study was that it utilized multiple data collection techniques and sources, which allowed triangulation of the findings from both approaches [82]. A limitation was that the data were collected in 2011. However, current literature suggests that the same challenges in infant feeding in the context of HIV persist and are still relevant [37, 70, 72, 77, 78]. There was also the possibility of influence on the roles of the interviewers as health care workers in KCH, which may have contributed to the reported ‘positive’ effect of interacting with the health system by mothers on adherence to infant feeding guidance [83].

## Conclusions

Infant feeding decision-making by mothers living with HIV is constrained by a lack of autonomy, stigma and poverty. Strategies that foster empowerment and psychosocial support of mothers, and increase the involvement of influential family members in infant feeding counseling sessions, would serve to optimize their infant feeding choices. HIV services should seek to embed themselves in other child health promotion services, to normalize infant feeding strategies in the context of HIV. Continuing professional development of health care workers should also be prioritized.

## Additional files

Additional file 1: Kiswahili version of observation tool. (DOCX 19 kb)

Additional file 2: Kiswahili version of focus group discussion guide. (DOC 49 kb)

## Abbreviations

AFASS: Acceptable feasible affordable sustainable and safe
ANC: Antenatal clinic
EID: Early Infant Diagnosis
FGDs: Focus group discussions
HCP: HIV clinic patient
HCWs: Healthcare workers
HIV: Human immunodeficiency virus
IDIs: In-depth interviews
KCH: Kilifi County Hospital
KCR: KEMRI Community Representatives
KEMRI: Kenya Medical Research Council
LAZ: Length for a z score
M2 M: Mother to mother
MAM: Moderate acute malnutrition
MCH: Maternal and child health clinic
MUAC: Mid upper arm circumference
NASCOP: National AIDS and Sexually transmitted disease Control Programme
PCR: Polymerase chain reaction
PNC: Postnatal clinic
SAM: Severe acute malnutrition
WHO: The World Health Organization
WLZ: Weight for length z score
