# Supplementary material for: What influences feeding decisions for HIV-exposed infants in rural Kenya?
Source: Int Breastfeed J. 2017 Jul 12;12:31. doi: 10.1186/s13006-017-0125-x (PMC5508793; doi:10.1186/s13006-017-0125-x)
Supplement: Supplementary file 1 — Kiswahili version of observation tool. (DOCX 19 kb) [file 13006_2017_125_MOESM1_ESM.docx]

**MADA ZITAKAZO JADILIWA KWENYE VYOMBO VYA UTAFITI WA UBORA**

Uchunguzi wa rasmi na nusu-muundo, mahojiano ya kina na maongezi ya makundi yataongozwa na Mwana soshologia mwenye uzoefu wa kutekeleza mbinu hizi za ukusanyaji wa ujumbe akisaidiwa na Mhudumu wa nyanjani anaye jua kuongea na kuandikika lugha asili. Vipengele vitakavyo jadiliwa vitaendelezwa zaidi kadri utafiti huu utakavyo kuwa ukiendelea.

**Muongozo wa uchunguzi wa rasmi na nusu-muundo**

Hizi zitanuiwa kupeana taswira ya vile ushauri wa lishe unatekelezwa na wahudumu wa afya toufauti tofauti. Sehemu zenye umuhimu ni kama:

**1. Mahali ambapo ushauri unatekelezwa**

- Ni nani anapeana ujumbe? Nambari na kiwango cha wahudumu?

- Ni wapi ujumbe huu unapeanwa?
- Je, ni mahali palipo wazi na kuna shughuli zingine ambazo zinaendelea ama ni kwenye chumba kisicho na kelele?
- Ni nani anapewa ujumbe? Ni ushauri wa jumuia (makundi) ama ni wa kibinafsi?

- Kuna usumbufu/kuingia na kutoka wakati ushauri unaendelea?

**2. Vihusishi na mbinu za kutoa ujumbe**

- Je, ujumbe unapeanwa kwa lugha inayo eleweka na mzazi/mlezi?

- Je, ni sawia na kiwango chake cha elimu?
- Je, ujumbe unaopenwa na wahudumu wa afya ni sahihi kama ulivyo kwenye taratibu zinazo husu lishe ya watoto Nnchini (Kenya) na zile za ulimwengu (W.H.O)?
- Je, wahudumu wa afya wanapeana aina moja ya ujumbe katika mazingara tofauti tofauti? k.v. katika kliniki ya akinamama walio jifungua, kliniki ya jamii (CCRC).
- Kwa ujumla, ni muda gani hutumika wakati wa matembezi ya kliniki?
- Ni muda gani hutumika wakati wa ushauri kuhusu lishe katika matenbezi ya kliniki?
- Je, ni wakati gani wa matembezi ambapo ushauri wa lishe hujadiliwa.
- Je, kuna kuhakikisha kwamba mama/mlezi ameelewa kuhusu maelezo muhimu? Ikiwa ndio, ni kivipi?
- Je, mama/mlezi hupata wakati wa kuuliza mwaswali? Ikiwa ni ndio, Je, anauliza; na maswali haya yanakabiliwa vipi?
- Je mzazi/mlezi anapewa ujumbe wakuandikwa/anaonyeshwa picha za mbinu bora za lishe ya mtoto?

**3.MIENENDO YA UINGILIANO KATI YA WAHUDUMU NA WALEZI/WAZAZI**

- Matumizi ya ishara kama vile unavyo keti/kusimama na aina ya mavazi
- Je wahudumu wanakaribika? Inajitokeza kwa juhudi kama vile,kuwaweka kinamama/walezi katika hali isiyo ya taharuki baada ya ushauri kwa kuangalia ujumbe wanao hitaji, wameelewa vipengele muhimu na kuwaruhusu wateja wao kuwaendea wakati wowote wakiwa na maswali/tashwishi.

**Taratibu kuhusu mahijiano ya kibinafsi na majadiliano ya makundi**

**Mahojiano ya kina (na wazazi/walezi walioathirika na walio na watoto wa umri chini ya miaka 2 pamoja na wazazi kama hawa ambao hali yao kuhusiana na virusi haijulikani) .**

- Maoni ya kibinafsi kuhusu lishe ya watoto
- Maoni ya kibinafsi kuhusu lishe ya watoto katika swala lihusianalo na maambukizo ya virusi kwa mama na ama kwa mtoto.
- Kuzimbua taratibu kuhusu lishe ya watoto walio katika hathari ya maambukizo ya virusi ama ambao tayari wameshaambukizwa na wametambulika kwamba wanapata lishe nzuri na kuendendelea vyema pamoja na wale walio na ukosefu wa kiasi/mwingi wa lishe.
- Kuzimbua maoni ya jumla kwa wahusika kuhusu ushauri wa lishe ya watoto unaopeanwa hospitalini.
- Je, ni ujumbe gani wanao kumbuka kutokana na ushauri kuhusu lishe waliopata?
- Ni yapi kati ya ujumbe huu ambao walikuwa hawajawahi kuyasikia, yalikuwa mageni/ya kuwashangaza?
- Kuzimbua ikiwa na ni vipi ujumbe huu umekuwa ukitumika nyumbani
- Ni vizuizi gani hukumba wahudumu ikitokea kwamba ujumbe waliopeana haukutekelezwa majumbani?
- Kuzimbua vikwazo vya uzingatiaji wa taratibu za lishe zilizopendekezwa kwa watoto walio katika athari ya maambukizo ama ambao tayari wamepata maambukizo ya virusi, haswa kwa heshima ya kuendeleza unyonyeshaji watoto pekee.
- Ni vyanzo gani vya habari /ushauri ambavyo mama yuko navyo (k.v. nyanya, jamaa, majirani,makundi ya akina mama)?
- Kuzimbua sababu zinazo changia utekelezaji mwema wa ushauri kuhusu lishe ya mtoto.

**Maongezi ya makundi na wazazi/walezi walioathirika na virusi ambao wamejiunga na vikundi na wale wazazi ambao hawajajua hali zao kuhusu virusi ama hawajapata maambukizo pamoja na wawakilishi wa jamii wa KEMRI. Maswali haya yatalenge majibu ya mahojiano na yaliyo onekana lakini yatajumuisha:**

- Kuzimbua zaidi maoni ya jamii kuhusu lishe ya watoto katika swala linalo/lisilo husu maambukizo ya virusi
- Kuzimbua itikadi za kijamii na matarajio yao kwa walezi tukizingingatia swala kuhusu lishe ya watoto.
- Kuzimbuz vikwazo vya lishe toshelezi katika swala linalo/lisilo husu maambukizo ya virusi.
- Kuzimbua maoni ya jamii kuhusu ushauri wa lishe ya watoto unaopeanwa hospitalini

**Maongezi ya makundi na wahudumu wa afya**

- Mafakirio kuhusu lishe ya watoto haswa katika swala linalo husu maambukizo ya virusi kwa mama na ama kwa mtoto.
- Vyanzo vya ujuzi na nafasi za mafundisho kuhusu ushauri wa lishe kwa watoto
- Kuzimbua mafikirio kuhusu hathari za maambukizo ya virusi kupitia kwa maziwa ya titi na maoni dhidi ya wahudumu wa afya kuhusu mikakati ya lishe kwa watoto katikaswala linalohusu virusi kwa hivi vituo tofauti.
- Kuzimbua changamoto wanazo kumanbana nazo wakati wakupeana ushauri wa lishe ya watoto haswa katika swala linalo husu virusi na mapendekezo jinsi vile kila changamoto inaweza kutatuliwa.
