# Supplementary material for: What influences feeding decisions for HIV-exposed infants in rural Kenya?
Source: Int Breastfeed J. 2017 Jul 12;12:31. doi: 10.1186/s13006-017-0125-x (PMC5508793; doi:10.1186/s13006-017-0125-x)
Supplement: Supplementary file 2 — Kiswahili version of focus group discussion guide. (DOC 49 kb) [file 13006_2017_125_MOESM2_ESM.doc]

**MUONGOZO WA MAHOJIANO YA MAKUNDI KWA WALEZI WA KIKE WALIO ATHIRIKA NA WALE AMBAO HAWAJA ATHIRIKA NA VIRUSI VYA UKIMWI (HIV)**

**Utangulizi**

Majina yetu ni: HN, RA, CK. Kama moja wapo ya tafiti endelezi kutusaidia kujua zaidi kuhusu mpangilio wa lishe kwa watoto wachanga katika jamii: tungependelea kusoma zaidi kuhusu jinsi vile watoto wachanga wanavyo pewa lishe katika hii jamii.haswa wakati mama ameathirika na virusi na nichangamoto gani zinazo wakumba nyinyi kama walezi ili kwamba mkaweze kuchangia maoni kuhusiana na vile taratibu muafaka zitakavyoweza chukuliwa na kama ipasavyo kwa watu wa eneo hili.  **Maswali**

**Maamuzi**

- Ni kwa jinsi gani akinamama hufanya uamuzi wa kawaida juu ya, ni aina gani ya vyakula vya watawawapatia watoto katika miezi sita ya kwanza?How
- Ni wakati gani akina mama hufanya uamuzi wa kawaida kuhusu aina za chakula watakacho peana? Je ni kabla ama baada ya kuzaa? Je itategemea na iwapo ni mzaliwa wa kwanza au la? Ni kwa jinsi gani maambukizo ya virusi huathiri hili? K.v. Ni wakati gani uamuzi hufanywa? Na ni uamuzi gani?
- Ni nini ama ni nani huchangia maamuzi kuhusiana na aina za lishe (kwa akina mama walio athirika na Virusi)? Na ni kivipi? Ni nini hukufanya wewe kuchagua yale utakayo yafanya?
- Ni nani hushirikishwa (endapo hufanyika)? Je, ni Bwana/Wapenzi? Akina mama mkwe? Akina mama wazazi? Ama Marafiki? Je kuhusika kwao ni kwa msaada? Je unapata msaada wowote wa kukuwezesha kufanya uamuzi? Je akina mama hujiona ya kwamba wao ndio waliofanya uamuzi ama ni kitu ambacho huwatokea tu?
- Je, ni ujumbe wa mahali pengine nnje katika jamii? Ama ni ujumbe wa hospitali? Ni ujumbe gani hupeanwa kwa akina mama katika hospitali kuhusu ulishaji wa watoto?Je ujumbe huu ni wa msaada? Je ni ujumbe gani ulio wa msaada zaidi na ni kwa nini?
- Je ni ujumbe gani ulio wa msaada haba na ni kwa nini? (k.m. sio mrahisi kutekelezwa na waamuzi walio tajwa mwanzoni? K.v. Ni ujumbe unaokaganya). Je ni taarifa gani ambayo imeweza kubadilisha mazowea yako ya ulishaji watoto? Ni kwa nini na ni kivipi?
- Je kuna wowote kati ya ushauri unao peanwa ambao ni wa utata ama wa mgongano? Ni kwa nini na ni vipi huwa ni wa mgongano?

**Mazoeya ya ulishaji**

- Tumekuwa tukisikia kwamba lishe mchanganyiko imekubalika kijamii kuliko ile lishe ya maziwa ya titi la mama pekee barani Africa..Je swala kama hili hufanyika hapa?We have often heard that mixed feeding is more culturally acceptable than exclusive breastfeeding in Africa…is this the case here?
- Pia tumesikia ya kwamba baadhi ya akina mama Africa wameweza kupeana tu maziwa ya titi kwa watoto wao katika miezi sita ya kwanza...Je swala kama hili hufanyika hapa??
- Je ni aina gani za chakula hupeanwa ili kuongezea maziwa ya titi la mama katika miezi sita ya kwanza? Ni kitu gani humsaidia mama kufanya uwamuzi wa kuhusu wakati wa kumuanzisha k.v. ‘uji**’**

**athari zinazotarajiwa**

- Je kuna manufaa ama hasara gani kwa mtoto aliye amuliwa kunyonyeshwa tu katika miezi sita ya kwanza? Je kuna manufaa ama hasara gani kwa mama aliye amua kunyonyesha tu katika miezi sita ya kwanza?

***Maambukizo ya virusi vya ukimwi***

- Ni lipi kati ya yaliyo tajwa huchangiwa na kuathirika na Virusi vya Ukimwi kwa mama?
- Ni jambo gani unalolifahamu kuhusu usambazaji Virusi kutoka kwa mama hadi kwa mwanawe? Je inaweza kufanyika? Kivipi?
- Endapo mama ameathika, ni athari kiwango gani kwamba atamwambukiza mwanawe endapo ataamua kumpatia mwanawe maziwa ya titi tu?

**Mikakati ya kukabiliana**

- Hisikua wahudumu wengi wakisema “kunyonyesha tu” ni nini haswa maana yake? Na maji je? Tunajua ya kwamba, “kunyonyesha tu bila maji” katika miezi sita ya kwanza huwa na changamoto kubwa. Je,kuna shinikizo na change moto gani? Katika siku za mwanzo? Na baada ya miezi ya mwanzo?
- Je akina mama wanao fanya, huweza vipi? Je hukabili vipi shinikizo? Je Mabwana zao huunga mkono swala la “kunyonyesha tu”? je ni kwa njia gani? Ni vipi kuhusu jamaa zao wa kike na marafiki? Ama yeyote yule? Je “kunyonya to” ni mbinu ya kikweli katita mazingara haya.
- Je kuna changamoto gani pale mwanzoni unapoanza kumpa mtoto vyakula vigumu katika hii jamii k.v. uji? Kwa akina mama waliio athirika na Virusi? Na viipi mnavyo ikabili?

## Maoni kuhusu ujumbe ama mabadiliko ambayo yatakuwa na umuhimu kwenye mashauri hapa.

## una maoni yoyote? Je kuwaalika akina baba katika vikao vya mashauri ya lishe kwa watoto kutakuwa na usaidizi wowote?

## Maswali yasionuiwa

- kwa ujumla, mankuli hugawanywa vipi kati ya familia? K.v. akina baba, akina nyanya/babu na watoto.
- Je mpangilio wa umuhimu huwa vipi katitka majumba huswa likizingatiwa swala kuhusu lishe? Je ni baba ama mtoto. Je ni nini hufanyika kukiwa na uhaba wa pesa?
- Endapo ungekuwa na raslimali haba, ungezitumia vipi kumlisha mtoto wako?.. ni kitu gani kinakuzuia kufanya hivi.......

*Je elimu ya maelezo inasaidia katika kufanya uamuzi wa mikakati kuhusu lishe ya watoto?iweke katika kunyonyesha tu/ linganisha na makaazi ya mijini pia.*
